# Minimum Free Energy Path of Ligand-Induced Transition in Adenylate Kinase

**DOI:** 10.1371/journal.pcbi.1002555

**Published:** 2012-06-07

**Authors:** Yasuhiro Matsunaga, Hiroshi Fujisaki, Tohru Terada, Tadaomi Furuta, Kei Moritsugu, Akinori Kidera

**Affiliations:** 1 Computational Science Research Program, RIKEN, Wako, Japan; 2 Special Postdoctoral Researchers Program, RIKEN Advanced Institute for Computational Science, Chuo-ku, Kobe, Japan; 3 Department of Physics, Nippon Medical School, Nakahara, Kawasaki, Japan; 4 Agricultural Bioinformatics Research Unit, Graduate School of Agricultural and Life Sciences, The University of Tokyo, Bunkyo-ku, Tokyo, Japan; 5 Graduate School of Bioscience and Biotechnology, Tokyo Institute of Technology, Midori-ku, Yokohama, Japan; 6 Graduate School of Nanobioscience, Yokohama City University, Yokohama, Japan; Stanford University, United States of America

## Abstract

Large-scale conformational changes in proteins involve barrier-crossing transitions on the complex free energy surfaces of high-dimensional space. Such rare events cannot be efficiently captured by conventional molecular dynamics simulations. Here we show that, by combining the on-the-fly string method and the multi-state Bennett acceptance ratio (MBAR) method, the free energy profile of a conformational transition pathway in *Escherichia coli* adenylate kinase can be characterized in a high-dimensional space. The minimum free energy paths of the conformational transitions in adenylate kinase were explored by the on-the-fly string method in 20-dimensional space spanned by the 20 largest-amplitude principal modes, and the free energy and various kinds of average physical quantities along the pathways were successfully evaluated by the MBAR method. The influence of ligand binding on the pathways was characterized in terms of rigid-body motions of the lid-shaped ATP-binding domain (LID) and the AMP-binding (AMPbd) domains. It was found that the LID domain was able to partially close without the ligand, while the closure of the AMPbd domain required the ligand binding. The transition state ensemble of the ligand bound form was identified as those structures characterized by highly specific binding of the ligand to the AMPbd domain, and was validated by unrestrained MD simulations. It was also found that complete closure of the LID domain required the dehydration of solvents around the P-loop. These findings suggest that the interplay of the two different types of domain motion is an essential feature in the conformational transition of the enzyme.

## Author Summary

Conformational transitions of proteins have been postulated to play a central role in various protein functions such as catalysis, allosteric regulation, and signal transduction. Among these, the relation between enzymatic catalysis and dynamics has been particularly well-studied. The target molecule in this study, adenylate kinase from *Escherichia coli*, exists in an open state which allows binding of its substrates (ATP and AMP), and a closed state in which catalytic reaction occurs. In this molecular simulation study, we have elucidated the atomic details of the conformational transition between the open and the closed states. A combined use of the path search method and the free energy calculation method enabled the transition pathways to be traced in atomic detail on micro- to millisecond time scales. Our simulations revealed that two ligand molecules, AMP and ATP, play a distinctive role in the transition scenario. The specific binding of AMP into the hinge region occurs first and creates a bottleneck in the transition. ATP-binding, which requires the dehydration of an occluded water molecule, is completed at a later stage of the transition.

## Introduction

Biological functions of proteins are mediated by dynamical processes occurring on complex energy landscapes [1]. These processes frequently involve large conformational transitions between two or more metastable states, induced by an external perturbation such as ligand binding [2]. Time scales of the conformational transition are frequently of order microseconds to seconds. To characterize such slow events in molecular dynamics (MD) trajectories, the free energy profile or the potential of mean force (PMF) along a reaction coordinate must be identified. In particular, the identification of the transition state ensemble (TSE) enables the barrier-height to be evaluated, and the correct kinetics would be reproduced if there is only a single dominant barrier. However, for proteins with many degrees of freedom, finding an adequate reaction coordinate and identifying the TSE is a challenging task placing high demands on computational resources.

The finite-temperature string method [3], [4], and the on-the-fly string method [5] find a minimum free energy path (MFEP) from a high-dimensional space. Given a set of collective variables describing a conformational change, the MFEP is defined as the maximum likelihood path along the collective variables. The MFEP is expected to lie on the center of reactive trajectories and contains only important transitional motions [4]. Furthermore, since the MFEP approximately orthogonally intersects the isocommittor surfaces (the surfaces of constant committor probability in the original space) [4], the TSE can be identified as the intersection with the isocommittor surface with probability of committing to the product (or the reactant) = 1/2. The methods and MFEP concepts have been applied to various molecular systems [4]–[6] including protein conformational changes [7]–[9].

With regard to high-dimensional systems like proteins, the quality of the MFEP (whether it satisfies the above-mentioned properties) is particularly sensitive to choice of the collective variables. The collective variables should be selected such that their degrees of freedom are few enough to ensure a smooth free energy surface; at the same time they should be sufficiently many to approximate the committor probability [4], [9]. To resolve these contrary requirements, effective dimensional reduction is required. Large conformational transitions of proteins, frequently dominated by their domain motions, can be well approximated by a small number of large-amplitude principal modes [2], [10]. This suggests that the use of the principal components may be the best choice for approximating the committor probability with the fewest number of variables for such large conformational transitions involving domain motions. A further advantage is the smoothness of the free energy landscape in the space of the large-amplitude principal components. If the curvature of the MFEP is large, the MFEP may provide a poor approximation to the isocommittor surface since the flux can occur between non-adjacent structures along the path [9]. The selection of the large-amplitude principal components as the collective variables would maintain the curvature of the MFEP sufficiently small.

Here, we conducted preliminary MD simulations around the two terminal structures of the transition and performed a principal component analysis to obtain the principal components (see *Materials and Methods* for details).

Following selection of a suitable MFEP, determination of the PMF and characterization of the physical quantities along the MFEP are needed to understand an in-depth mechanism of the transition. Although the finite-temperature string method yields a rigorous estimate of the gradient of the PMF under a large coupling constant with the collective variables [3]–[5] (see *Materials and Methods*), errors in the estimates of the gradients and in the tangential directions of the pathway tend to accumulate during the integration process. To accurately quantify the PMF and the averages of various physical quantities in a multi-dimensional space, we utilized another statistical method, the multi-state Bennett acceptance ratio (MBAR) method [11], which provides optimal estimates of free energy and other average quantities along the MFEP.

**Figure 1:**
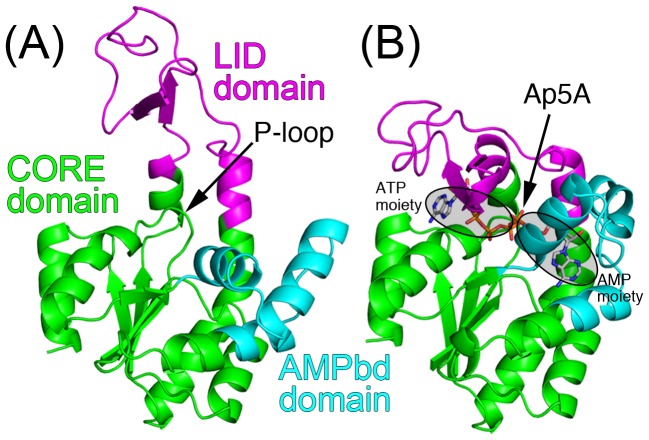
Crystal structures of *E. coli* AK. (A) Open conformation without ligand (PDBid: 4ake). The position of P-loop is indicated. (b) Closed conformation with Ap5A represented by sticks (PDBid: 1ake). The ATP and AMP moieties are encircled. Three relatively rigid domains, designated CORE, AMPbd, and LID, are colored by green, cyan, and magenta, respectively.

Here, we applied the above proposed methods to the conformational change in *Escherichia coli* adenylate kinase (AK), the best-studied of enzymes exhibiting a large conformational transition [12]–[23]. AK is a ubiquitous monomeric enzyme that regulates cellular energy homeostasis by catalyzing the reversible phosphoryl transfer reaction: ATP+AMP↔2ADP. According to the analysis of the crystal structures by the domain motion analysis program DynDom [24], AK is composed of three relatively rigid domains (Figure 1); the central domain (CORE: residues 1–29, 68–117, and 161–214), an AMP-binding domain (AMPbd: 30–67), and a lid-shaped ATP-binding domain (LID: 118–167). Inspection of the crystal structures suggests that, upon ligand binding, the enzyme undergoes a transition from the inactive open form to the catalytically competent closed structure [25] (Figure 1). This transition is mediated by large-scale closure motions of the LID and AMPbd domains insulating the substrates from the water environment, while occluding some catalytically relevant water molecules. The ATP phosphates are bound to the enzyme through the P-loop (residues 7–13), a widely-distributed ATP-binding motif.

The interplay between AK's dynamics and function has been the subject of several experimental studies. ^15^N NMR spin relaxation studies have revealed that the LID and AMPbd domains fluctuate on nanosecond timescales while the CORE domain undergoes picosecond fluctuations [12], [13]. The motions of these hinge regions are highly correlated with enzyme activity [14]. In particular, the opening of the LID domain, responsible for product release, is thought to be the rate-limiting step of the catalytic turnover [14]. Recent single-molecule Förster resonance energy transfer (FRET) experiments have revealed that the closed and open conformations of AK exist in dynamic equilibrium even with no ligand present [15], [16], and that the ligand's presence merely changes the populations of open and closed conformations. This behavior is reminiscent of the population-shift mechanism [26] rather than the induced-fit model [27], in which structural transitions occur only after ligand binding.

The population-shift like behaviour of AK has also been supported by simulation studies [17]–[20]. Lou and Cukier [17], Kubitzki and de Groot [18], and Beckstein *et al.* [19] employed various enhanced sampling methods to simulate ligand-free AK transitions. Arora and Brooks [20] applied the nudged elastic band method in the pathway search for both ligand-free and ligand-bound forms. These studies showed that, while the ligand-free form samples conformations near the closed structure [17]–[20], ligand binding is required to stabilize the closed structure [20].

Despite the success of these studies based on all-atom level models, atomistic details of the transition pathways, including the structures around the TSE, have not been fully captured yet. In this study, we successfully evaluated the MFEP for both ligand-free and ligand-bound forms of AK using the on-the-fly string method, and calculated the PMF and the averages of various physical quantities using the MBAR method. Our analysis elucidates an in-depth mechanism of the conformational transition of AK.

## Results

### Minimum Free Energy Path and the Corresponding Domain Motion

**Figure 2:**
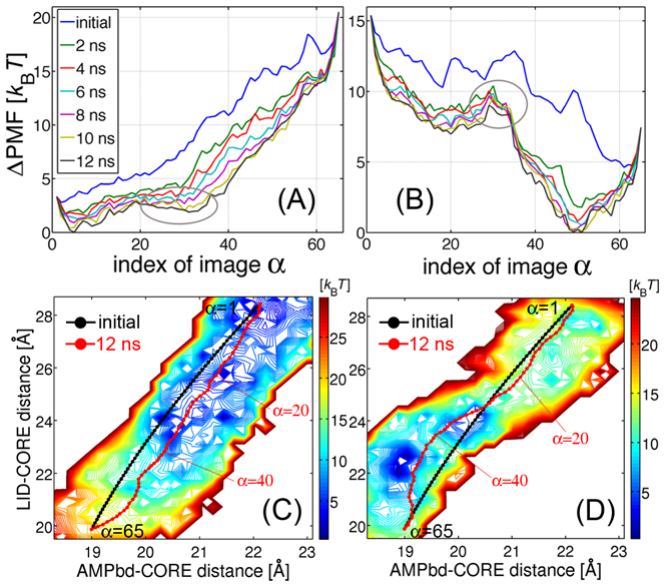
PMF along the strings and the corresponding domain motions. (Top) PMFs along the snapshots of the strings for (A) apo and (B) holo-AK at *t* = 0 (initial path), 2, 4, 6, 8, 10, 12 (MFEP) ns. The indices of string images are numbered from the open (PDBid: 4ake) to closed crystal structures (PDBid: 1ake). Partially-closed state of apo-AK and a substantial PMF barrier of holo-AK are encircled. (Bottom) Projections of MFEP onto the space defined by the distances of 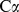 mass centers between the LID-CORE and AMPbd-CORE domains, for (C) apo and (D) holo-AK. The black curves indicate the initial paths (*t* = 0), and the red curves are the MFEPs (*t* = 12 ns). The PMF is visualized by the colored contour lines (delineating regions of low energy (blue) to regions of high energy (red)).

The MFEPs for apo and holo-AKs, and their PMFs, were obtained from the string method and the MBAR method, respectively (see Videos S1 and S2). The MFEPs were calculated using the same 20 principal components selected for the collective variables. The holo-AK calculations were undertaken with the bisubstrate analog inhibitor (Ap5A) as the bound ligand without imposing any restraint on the ligand. Figures 2A and 2B show the MBAR estimates of the PMFs along the images of the MFEP (the converged string at 12 ns in Figures 2A and 2B) for apo and holo-AK, respectively. Here, the images on the string are numbered from the open (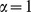; PDBid: 4ake [28]) to the closed conformation (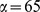; PDBid: 1ake [29]). These terminal images were fixed during the simulations to enable sampling of the conformations around the crystal structures. In the figures, the convergence of the PMF in the string method process is clearly seen in both systems. Convergence was also confirmed by the error estimates (Figure S1), and by the root-mean-square displacement (RMSD) of the string from its initial path (Figure S2). The PMF along the MFEP reveals a broad potential well on the open-side conformations of apo-AK, suggesting that the open form of AK is highly flexible [20]. This broad well is divided into two regions, the fully open (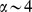) and partially closed states (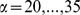, encircled) by a small PMF barrier. In holo-AK (Figure 2B), the MFEP exhibits a single substantial free energy barrier (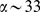) between the open and closed states, which does not appear in the initial path. This barrier will be identified as the transition state below. It is shown in the PMF along the MFEP that the closed form (tightly binding the ligand) is much more stable than the open form with loose binding to the ligand (large fluctuations of the ligand will be shown later).

To characterize the MFEP in terms of the domain motions, the MFEP was projected onto a space defined by two distances from the CORE domain, the distance to the LID domain and the distance to the AMPbd domain (the distance between the mass centers of 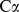 atoms for the two domains; Figures 2C and 2D). The PMF was also projected onto this space. The comparison of the two figures shows that ligand binding changes the energy landscape of AK, suggestive that this is not a simple population-shift mechanism. In apo-AK, the motions of the LID and AMPbd domains are weakly correlated, reflecting the zipper-like interactions on the LID-AMPbd interface [19]. The MFEP clearly indicates that the fully closed conformation (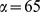) involves the closure of the LID domain followed by the closure of the AMPbd domain. The higher flexibility of the LID domain has been reported in previous studies [17], [19], [20]. In holo-AK, the pathway can be described by two successive scenarios, that is, the LID-first-closing followed by the AMPbd-first-closing. In the open state (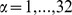), the MFEP is similar to that of apo-AK, revealing that LID closure occurs first. In the closed state (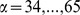), however, the AMPbd closure precedes the LID closure. This series of the domain movements was also identified by the domain motion analysis program DynDom [24] (Figure S3).

It is known in the string method that the convergence of the pathway is dependent on the initial path. In order to check whether the MFEP obtained here is dependent on the initial path or not, we conducted another set of the calculations for apo-AK by using a different initial path, which has an AMPbd-first-closing pathway, opposed to the LID-first-closing pathway shown above. If the LID and AMPbd domains move independently of each other, it is expected that LID-first-closing and AMPbd-first-closing pathways are equally stable. Despite this initial setup, however, our calculation again showed the convergence toward the LID-first-closing pathway (see Figure S4). As described above, this tendency of the pathways would be due to the reflection of the highly flexible nature of the LID domain.

Furthermore, in order to check whether the samples around the MFEP are consistent with the experiments, we compared the PMF as a function of the distance between the Cα atoms of Lys145 and Ile52 with the results of the single-molecule FRET experiment by Kern et al. [16] (see Figure S5). The PMF was calculated by using the samples obtained by the umbrella samplings around the MFEP. In the figure, the stable regions of the PMF for holo-AK are highly skewed toward the closed form, and some population toward the partially closed form was also observed even for apo-AK, which is consistent with the histogram of the FRET efficiency [16].

### “Cracking” in the Partially Closed State in apo-AK

**Figure 3:**
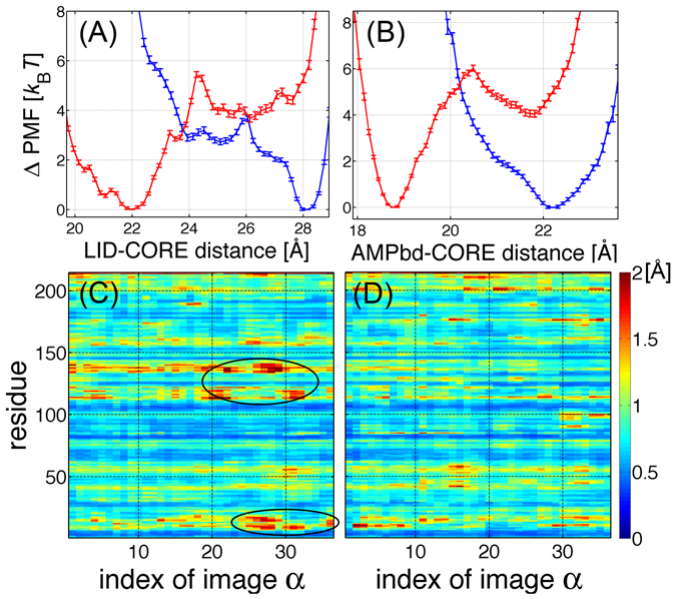
PMFs of the domain motions and the RMSF along the MFEP. (Top) PMF along the MFEP as a function of the inter-domain distances between (A) the LID-CORE and (B) AMPbd-CORE domains. The blue and red curves illustrate the results for apo-AK and holo-AK, respectively. The error bars are the statistical uncertainties relative to the PMF minimum. Note that the uncertainties are small because the domains were restricted to the regions around the MFEP. (Bottom) The RMSF of the 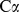 atoms along the MFEP is shown as a function of the MFEP images for (C) apo and (D) holo-AK. The “cracked” regions with large RMSF values, around the LID-CORE hinge and P-loop, are encircled.

To more clearly illustrate the energetics along the MFEP in terms of the domain motions, we separately plot the PMF as a function of the two inter-domain distances defined above (Figures 3A and 3B). We observe that the PMF of apo-AK has a double-well profile for the LID-CORE distance (indicated by the blue line in Figure 3A), whereas the PMF in terms of the AMPbd-CORE distance is characterized by a single-well (Figure 3B). The single-molecule FRET experiments monitoring the distances between specific residue pairs involving the LID domain (LID-CORE (Ala127-Ala194) [15] and LID-AMPbd (Lys145- Ile52) [16]) revealed the presence of double-well profiles in the ligand-free form. On the other hand, an electron transfer experiment probing the distance between the AMPbd and CORE domains (Ala55-Val169) [30] showed only that the distance between the two domains decreased upon ligand binding. Considering the PMF profiles in the context of these experimental results, we suggest that the partially closed state (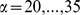) in apo-AK (Figure 2A) can be ascribed to the LID-CORE interactions but not to the AMPbd-CORE interactions.

To elucidate the origin of the stability of the partially closed state, we monitored the root mean square fluctuations (RMSF) of the 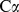 atoms along the MFEP (see *Materials and Methods* for details). Figures 3C and 3D show the RMSF along the MFEP for apo and holo-AK, respectively. In apo-AK (Figure 3C), large fluctuations occur in the partially closed state (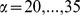) around the LID-CORE hinge regions (residue 110–120, and 130–140) and the P-loop (residue 10–20). It has been proposed, in the studies of AK using coarse-grained models, that “cracking” or local unfolding occurs due to localized strain energy, and that the strained regions reside in the LID-CORE hinge and in the P-loop [21], [23]. Our simulation using the all-atom model confirmed the existence of “cracking” in the partially closed state, and provided an atomically detailed picture of this phenomenon. The average structures around the partially closed state revealed that, in the open state, a highly stable Asp118-Lys136 salt bridge is broken by the strain induced by closing motion around 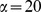 (Figure S6A). This salt bridge has been previously proposed to stabilize the open state while imparting a high enthalpic penalty to the closed state [18]. Breakage of the salt bridge releases the local strain and the accompanying increases in fluctuation may provide compensatory entropy to stabilize the partially closed state. A similar partially closed state of the LID domain was also found by the work of Lou and Cukier [31] in which they performed all-atom MD simulation of apo-AK at high temperature (500 K) condition.

In holo-AK, both of the LID-CORE and AMPbd-CORE distances exhibit double-well profiles (indicated by the red lines in Figures 3A and 3B), separating the closed from the open state. The breakage of the 118–136 salt bridge at around 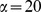 is not accompanied by “cracking” of the hinge region (Figure 3D). Instead, the hinge region is stabilized by binding of ATP ribose to Arg119 and His134 (Figure S6B), leading to a smooth closure of the LID domain. This suggests that one role of the salt bridge breakage is rearrangement of the molecular interactions to accommodate ATP-binding [32]. P-loop fluctuations are also suppressed in holo-AK (Figure 3D). Consistent with our findings, reduced backbone flexibilities in the presence of Ap5A were reported in the above-mentioned NMR study [13].

### Entropy Reduction and Misbinding of Ligand along the MFEP

**Figure 4:**
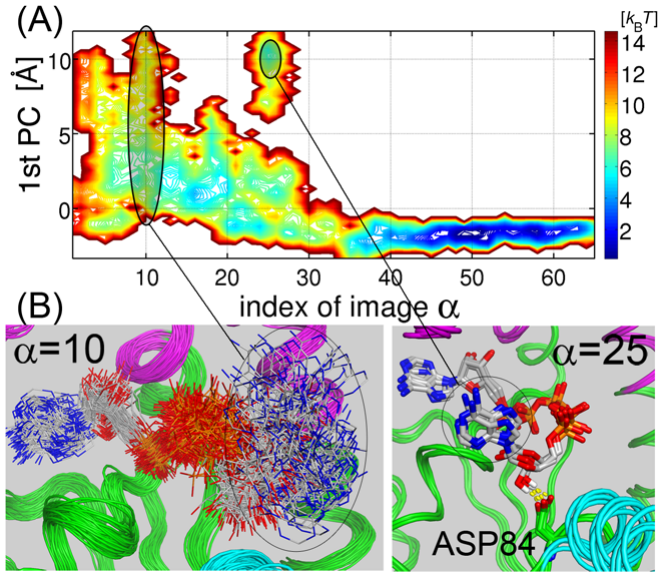
Two-dimensional PMF surface of the motion of the ligand along the MFEP. (A) PMF is projected onto the MFEP images and onto the 1st PC of the *xyz*-coordinates of the mass center of the AMP adenine. The PMF is represented by the colored contour lines. (B) Tens of the MD snapshots are superimposed at 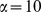 (left image), and 4 snapshots shows the misbinding between the AMP ribose and Asp84 at 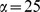 (right image). The hydrogen bonds are indicated by the dashed yellow lines.

The origin of the double-well profile in holo-AK was investigated via the ligand-protein interactions. The motion of the ligand along the MFEP was firstly analyzed by focusing on the AMP adenine dynamics, since the release of the AMP moiety from the AMP-binding pocket was observed in the open state. It is again emphasized that the ligand is completely free from any restraint during the simulations. PCA was performed for the three-dimensional Cartesian coordinates of the center of mass of AMP adenine, and the coordinates were projected onto the resultant 1st PC in Figure 4A. The AMP adenine is observed to move as much as 10 Å in the open state (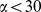; Figure 4B), while it is confined to a narrow region of width 1–2 Å (the binding pocket) in the closed state (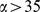). Such a reduction of the accessible space of the AMP adenine might generate a drastic decrease in entropy or an increase in the PMF barrier of the open-to-closed transition. Furthermore, close inspection of the PMF surface reveals the existence of a misbinding event at 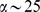 (Figure 4B), in which the AMP ribose misbinds to Asp84 in the CORE domain, and is prevented from entering the AMP-binding pocket. This event further increases the barrier-height of the transition.

### Highly Specific Binding of AMP Determines the Transition State Ensemble

**Figure 5:**
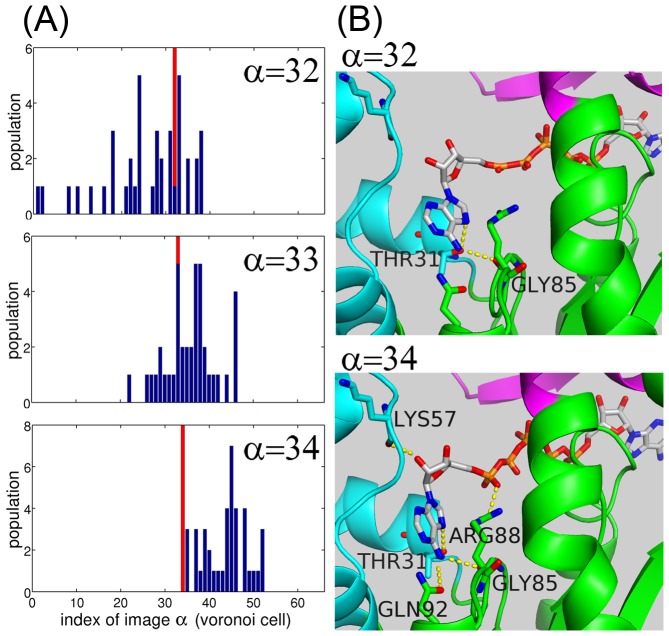
Committor tests characterizing the TSE. (A) The binned distributions of the final structures after 10 ns unrestrained MD simulations (blue bars), assigned by index of the nearest MFEP image (i.e., classified by the Voronoi tessellation). The MD simulations were executed from different initial distributions (red bars) at 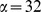, 33, and 34. (B) The average structures of the MFEP images at 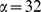 (before the TSE), and 34 (after the TSE). The ligand and the residues of Thr31, Lys57, Arg88, Gly85, and Gln92 are represented by sticks. The hydrogen bonds are indicated by the dashed yellow lines.

The MFEP revealed that AMP adenine enters the AMP-binding pocket around 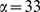, as indicated by a rapid decrease in the accessible area (Figure 4A). This event is well correlated with the position of the PMF barrier along the MFEP (Figure 2B). This coincidence between the binding process and the domain closure suggests that the two processes are closely coupled. Before analyzing the situation in detail, however, it is necessary to assess whether the observed PMF barrier around 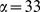 (Figure 2B) corresponds to a TSE, because the PMF barrier is not necessarily a signature of dynamical bottleneck in high-dimensional systems [33]. TSE validation is usually performed with a committor test [4], [7], [9], [33]. In principle, the committor test launches unbiased MD simulations from structures chosen randomly from the barrier region, and tests whether the resultant trajectories reach the product state with probability 1/2. Here, since limited computational resources precluded execution of a full committor test, 40 unbiased MD simulations of 10 ns were initiated from each of 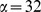, 33 or 34, a total of 120 simulations or 1.2 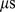, and the distributions of the final structures after 10 ns were monitored [9]. Figure 5A shows the binned distributions of the final structures assigned by index of the nearest MFEP image (the blue bars). When the simulations were initiated from the image at 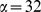 (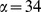), the distribution biases to the open form-side (the closed form-side) relative to the initial structures. On the other hand, when starting from the image at 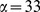, the distribution is roughly symmetric around the initial structures. This result suggests that the TSE is located at 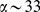. In other words, it was validated that the TSE was successfully captured in the MFEP, and at the same time, the collective variables were good enough to describe the transition.

A close inspection of the structures around the PMF barrier supported our insufficient committer test and revealed the mechanism of the ligand-induced domain closure. Figure 5B shows the hydrogen bond (H-bond) patterns between the ligand and the protein observed in the average structures at 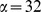 (before the TSE) and 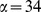 (after the TSE). At 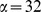, Thr31:OG1 (AMPbd) forms an H-bond with N7 of AMP adenine, and Gly85:O (CORE) forms one with adenine N6. These two H-bonds mediate the hinge bending of the AMPbd-CORE domains. In addition, the H-bond between Gly85:O and adenine N6 helps the enzyme to distinguish between AMP and GMP; GMP lacks an NH_2_ group in the corresponding position of AMP [34]. This means that the specificity of AMP-binding operates at an early stage of the ligand binding process. At 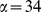, the AMPbd-CORE distance becomes smaller than that at 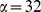, which allows the formation of 3 additional H-bonds with the ligand: Gln92:OE1 (CORE) and adenine N6, Lys57:O (AMPbd) and the ribose O2, and Arg88:NH1 (CORE) and O1 of AMP 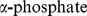. The resulting rapid enthalpy decrease stabilizes the closed conformation. Gln92:OE1 is also important in establishing AMP specificity; GMP lacks the counterpart atom, adenine N6. The strictly conserved Arg88 residue is known to be crucial for positioning AMP 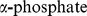 so as to suitably receive a phosphate group from ATP [35]. With regard to the AMPbd closure, our result suggests that Arg88 (CORE), in conjunction with Lys57 (AMPbd), works to block adenine release from the exit channel and to further compact the AMPbd-CORE domains.

### LID Domain Closure Follows the Dehydration Around the P-loop

**Figure 6:**
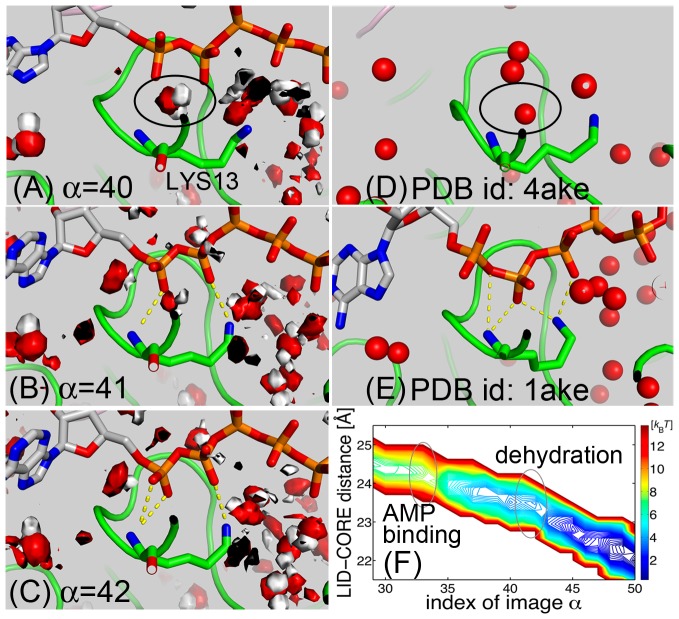
Dehydration of an occluded water around the P-loop. The isosurface representation of the 3D distribution function of water oxygen (red) and hydrogen (white) around the P-loop at (A) 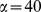, (B) 41, and (C) 42. The surfaces show the areas in which the atoms are distributed four times as probably as in the bulk phase. For comparison, the oxygens of crystal waters are shown for (D) the open (PDBid: 4ake) and (E) closed conformations (PDBid: 1ake). An occluded water molecule at 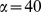 and the corresponding crystal water of the open form are indicated by the circles. (F) Two-dimensional PMF surface as a function of the MFEP images and the distances of the LID-CORE domains. The PMF is represented by the colored contour lines. Regions of physical events (AMP-binding and dehydration) are encircled.

A remaining question is how closure of the LID domain follows that of the AMPbd domain. Unlike the AMP-binding pocket, the ATP-binding sites, including the P-loop, are surrounded by charged residues, which attract interfacial water molecules. Upon LID closure, most of these water molecules will be dehydrated from the enzyme, but some may remain occluded. To characterize the behaviors of these water molecules, the 3D distribution function of their oxygen and hydrogen constituents were calculated along the MFEP using the MBAR method (see *Materials and Methods*). Figures 6A, 6B, and 6C display the isosurface representations of the 3D distribution functions around the P-loop at 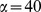, 41, and 42, respectively. The surfaces show the areas in which the atoms are distributed four times as probably as in the bulk phase. At 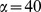, the ATP phosphates are not yet bound to the P-loop because an occluded water molecule (encircled) is wedged between the phosphate and the P-loop, inhibiting binding of ATP 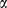 and 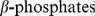 and bending of the side-chain of “invariant lysine” (Lys13), a residue that plays a critical role in orienting the phosphates to the proper catalytic position [36]. This occluded water molecule may correspond to that found in the crystal structure of apo-AK (PDBid: 4ake) (Figure 6D, encircled). Figures 6B and 6C clearly demonstrate that, upon removal of this water molecule, the ATP phosphates begin binding to the P-loop. These observations were confirmed by plots of the PMF surface mapped onto a space defined by the LID-CORE distance versus the index of image (Figure 6F), which shows that the PMF decreases discontinuously upon dehydration followed by LID domain closure. Interestingly, compared with the crystal structure (PDBid: 1ake)(Figure 6E), the position of the ATP moiety is shifted to the AMP side by one monophosphate unit. This may be a consequence of early binding of the AMP moiety. At a later stage (around 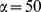), this mismatch was corrected to form the same binding mode as observed in the crystal structure. This reformation of the binding mode may be induced by the tight binding of ATP adenine to the LID-CORE domains, and will not occur in the real enzymatic system containing ATP and AMP instead of the bisubstrate analog inhibitor, Ap5A.

## Discussion

In this study, we have applied the on-the-fly string method [5] and the MBAR method [11] to the conformational change of an enzyme, adenylate kinase, and successfully obtained the MFEP (Figures 2A and 2B). The MFEP yielded a coarse-grained description of the conformational transitions in the domain motion space (Figures 2C and 2D). At the same time, the atomistic-level characterization of the physical events along the MFEP provided a structural basis for the ligand-binding and the domain motions (Figures 3–6). This kind of multiscale approach used here is expected to be useful generally for complex biomolecules since the full space sampling can be avoided in an efficient manner.

We have shown that in the TSE of holo-AK, the conformational transition is coupled to highly specific binding of the AMP moiety. Our results have been validated by unbiased MD simulations. The mechanism of the AMPbd domain closure is consistent with that proposed by the induced-fit model (Figure S7A), and follows a process similar to that of protein kinase A, previously investigated by a coarse-grained model [32]: (i) the insertion of the ligand into the binding cleft initially compacts the system; (ii) additional contacts between the ligand and non-hinge region further compact the system. The closure of the LID domain is more complicated (Figure S7B). It was shown that apo-AK can exist in a partially closed state, stabilized by the “cracking” of the LID-CORE hinge and the P-loop, even with no ligand present. The cracking of the hinge region enables rearrangement of molecular interactions for ATP-binding, which induces a smooth bending of the hinge. Along with the LID closure, ATP is conveyed into the P-loop, with removal of an occluded water molecule. The closure of the LID domain follows the “population-shift followed by induced-fit” scenario discussed in Ref. [37], in which a transient local minimum is shifted toward the closed conformation upon ligand binding. This two-step process of the LID domain closure is similar to the two-step mechanism reported in recent simulation studies of the Lysine-, Arginine-, Ornithine-binding (LAO) protein [38] and the maltose binding protein [39].

In holo-AK, AMPbd domain closure occurs early (at 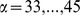), while the LID domain closes at later stages (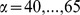). An interesting question is whether an alternative pathway is possible in the presence of the real ligands (ATP and AMP) instead of Ap5A. Ap5A artificially restrains the distance between the ATP and AMP moieties. During the process with real ligands, the dynamics of the LID and AMPbd domains is expected to be less correlated. Nevertheless, for full closing of the LID domain, we conjecture that the AMPbd domain should be closed first, enabling the interactions on the LID-AMPbd interface to drive the dehydration around the P-loop. This suggests that full recognition of ATP by the LID-CORE domains occurs at a later stage of the conformational transition. This conjecture may be related to the lower specificity of *E. coli* AK for ATP compared with AMP [34]. Nonspecific AMP-binding to the LID domain has previously been suggested to explain the observed AMP-mediated inhibition of *E. coli* AK at high AMP concentrations [40].

A missing ingredient in the present study is the quantitative decomposition of the free energy in each event, such as the ligand binding and the interactions on the LID-AMPbd interface. For enhanced understanding of the conformational change, our methods could be complemented by the alchemical approach [41]. Varying the chemical compositions of the system during the conformational change would enable us to elucidate the effects of ligand binding, cracking, and dehydration in a more direct manner.

## Materials and Methods

### Preparation of the System

We prepared three systems from the following initial structures: (i) “apo-open system”, X-ray crystal structure of the open-form without ligand (PDBid: 4ake [28]), (ii) “holo-closed system”, crystal structure of closed-form with Ap5A (PDBid: 1ake [29]), (iii) “apo-closed system”, structure created by removing Ap5A from the holo-closed system.

The protonation states of the titratable groups at pH 7 were assigned by PROPKA [42], implemented in the PDB2PQR program package [43], [44]. The apo-open and apo-closed systems yielded identical assignments, which were used also for the holo-closed system. These systems were solvated in a periodic boundary box of water molecules using the LEaP module of the AMBER Tools (version 1.4) [45]. A padding distance of 12 Å from the protein surface was used for the apo-open system. For the apo-closed and holo-closed systems, a longer padding distance of 20 Å was used to avoid interactions with periodic images during the closed-to-open transition. Two Na^+^ ions were added to neutralize the closed-apo and open-apo systems, while seven Na^+^ ions were required to neutralize the closed-holo system.

The systems were equilibrated under the NVT condition at 300 K by the following procedure: First, the positions of solvent molecules and hydrogen atoms of the protein (and Ap5A) were relaxed by 1,000 step minimization with restraint of non-hydrogen atoms. Under the same restraints, the system was gradually heated up to 300 K over 200 ps, followed by 200 ps MD simulation under the NVT condition at 300 K while gradually decreasing the restraint forces to zero, but keeping the restraints on 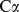 atoms needed in the string method. The system was further equilibrated by 200 ps MD simulation under the NPT condition (1 atm and 300 K), adjusting the density of the water environment to an appropriate level. The ensemble was finally switched back to NVT, and subjected to additional 200 ps simulation at 300 K, maintaining the 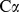 restraints.

The equilibration process was conducted using the Sander module of Amber 10 [45], with the AMBER FF03 force field [46] for the protein, and TIP3P for water molecules [47]. The parameters for Ap5A were generated by the Antechamber module of AMBER Tools (version 1.4) [45] using the AM1-BCC charge model and the general AMBER Force Field (GAFF) [48]. Covalent bonds involving hydrogen atoms were constrained by the SHAKE algorithm [49] with 2 fs integration time step. Long-range electrostatic interactions were evaluated by the particle mesh Ewald method [50] with a real-space cutoff of 8 Å. The Langevin thermostat (collision frequency 1 ps^−1^) was used for the temperature control.

The production runs, including the targeted MD, the on-the-fly string method, the umbrella sampling, and the committor test, were performed with our class library code 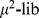 for multicopy and multiscale MD simulations (which will soon be available) [T. Terada et al., unpublished], using the same parameter set described above (unless otherwise noted). Protein structures and the isosurfaces of solvent density were drawn with PyMOL (Version 1.3, Schrödinger, LLC). The calculations were performed using the RIKEN Integrated Cluster of Clusters (RICC) facility.

### Principal Component Analysis and Setup of Initial Path

It has been shown that normal modes or principal modes provide a suitable basis set for representing domain motions of proteins [2], [10]. In particular, it has been argued that the conformational change in AK can be captured by a set of principal modes of apo-AK [31], [51]. In this study, we have defined the collective variables for the on-the-fly string method using the principal components of apo-AK. The PCA was carried out in the following manner: After the equilibration process, 3 ns MD simulations were executed at 300 K without restraint for both apo-open and apo-closed systems. The obtained MD snapshots from both systems were combined in a single PCA [52], removing the external contributions by iteratively superimposing them onto the average coordinates [53], [54]. The PCA was then conducted for the Cartesian coordinates of the 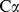 atoms. It was found that the first principal mode representing the largest-amplitude merely represents the difference between the open and closed conformations. The fluctuations in the two structures were expressed in the principal modes of smaller amplitudes. The cumulative contributions of these modes (ignoring the first) are shown in Figure S8. As expected, the principal modes represent the collective motions of the LID and AMPbd domains (Figure S9). The first 20 principal components (82% cumulative contribution, ignoring that of the first) were adopted as the collective variable of the string method. These components were sufficient to describe the motions of three domains in AK for which at least 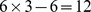 degrees of freedom are required in the rigid-body approximation. The additional eight degrees of freedom were included as a buffer for possible errors in the estimation of the principal modes. The sum of the canonical correlation coefficients between the two sets of the 20 principal components, one calculated using the samples of the first half (0–1.5 ns) snapshots and the other using the last half (1.5–3 ns) snapshots, was 11.8 (∼12), suggesting that the subspace of the domain motions was converged in 3 ns simulation.

The initial paths for the string method were generated using the targeted MD (TMD) simulations [55] for apo and holo-AK. Whereas a previous study had constrained the one-dimensional RMSD value from the starting structure to the target structure [55], in this study, we imposed 20-dimensional harmonic restraints (with spring constant 10 kcal/mol/Å^2^) along the linear interpolation between the open and closed crystal structures in the 20 principal component space [8], [9]. Starting from the closed conformation, TMD simulations of about 1 ns for the apo-closed and holo-closed systems were conducted with the open conformation as target. By imposing this direction, from the closed conformation to the open conformation, unfavorable steric crashes can be avoided. During the TMD, the Eckart condition [56] was imposed on the 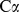 atoms. The initial path for the string was obtained as 65 structures on the TMD trajectory by equally partitioning the trajectory from the open to closed conformation. These 65 structures were equilibrated by 400 ps restrained MD simulations. Throughout this stage, the AMP moiety of the Ap5A separated from the AMP-binding pocket in the open state of the holo-closed system. However, the ATP phosphates remained bound to the correct side chains of the conserved arginines (Arg123, Arg156, and Arg167), and the ATP ribose was bound to the backbone of Arg119.

### On-the-Fly String Method

The on-the-fly string method [5], a variant of the original finite-temperature string method [3], is a powerful tool for finding the MFEP from high-dimensional free energy surfaces. The MFEP is searched on the free energy surface associated with *M* collective variables (or *M* ( = 20) principal components of 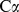 atoms in this case), 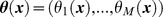, with ***x*** being the Cartesian coordinates of the entire system. The following equations are solved simultaneously:
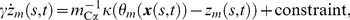
(1)

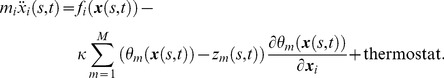
(2)Equation 1 describes the time evolution of the string 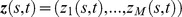 in the *M-*dimensional collective variable space, at position *s* on the string and at time *t* in the simulation. Equation 2 is a standard MD simulation with restraint of the collective variable 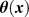 around 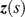. The parameter 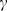 is the “friction” coefficient controlling the dynamics of 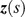, 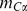 is the mass of 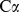 atom, and 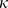 is a spring constant. For a proper choice of 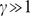 and 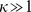, the string 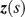 is driven by a negative gradient on the free energy surface and is expected to converge to the MFEP [5]. Here, we chose a strong spring constant of 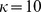 (kcal/mol/Å^2^) to reduce the statistical bias in the estimation of the free energy gradient while maintaining the numerical stability of the simulation. Also, 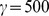 (kcal s/mol/Å) was chosen to reduce the statistical fluctuations of 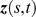 by slowing down the dynamics of 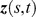 compared that of 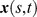. The position *s* on the string was discretized by 65 images, 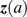 (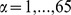), numbered from the open to the closed conformation. The terminal images were fixed to sample around the open and closed crystal structures. The term “constraint” in Equation 1 indicates that the distances between adjacent images, 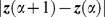, are kept equal through all 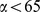, or 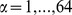 [4], [5]. It is noted that the principal components are based on the unitary transformation from the coordinates of the 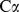 atoms to 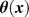. Thus, the metric tensor appearing in the original formulation [5] due to the curvilinear nature of the collective variables can be reduced from an 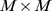 matrix as a function of ***x*** to a constant diagonal form 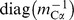. At the same time, two sets of the Cartesian coordinates of the entire system, 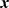 and 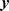, required for the statistical independence of the metric tensor in the original formulation [5], can also be reduced to the single variable 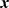. A further advantage of the principal components approach is that large-amplitude principal components may capture a smooth free energy surface and thus avoid the trapping of string images in local minima.

### MBAR Calculation with Umbrella Sampling and Reweighting

To accurately quantify the PMF and the averages of various physical quantities, the MBAR method [11], [57] was employed. The standard weighted histogram analysis method [58] requires an extremely large storage space for the grid points in the 20-dimensional space. The MBAR method requires no grid points and hence naturally circumvents this problem. Other advantages of the MBAR method are that the estimator is asymptotically unbiased and yields minimal variance, and that the statistical error can be estimated under the large sample limit [11]. In the following, we briefly summarize the umbrella sampling and the MBAR method.

The umbrella sampling was conducted around the image of the string obtained as the MFEP or at *t* = 12 ns. For comparison, umbrella sampling was also performed around the string images at *t* = 0, 2, 4, 6, 8, and 10 ns, generating a total of 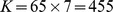 ensembles. The window potential 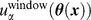 used for the sampling was the harmonic restraint imposed on the 20 principal components. Here, we chose a weak spring constant of 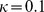 kcal/mol/Å^2^ to obtain sufficient phase space overlaps. Following a 200 ps equilibration, umbrella sampling was performed for 9 ns around each image of the MFEP. For the strings other than the MFEP, simulation time was limited to 0.8 ns to reduce the simulation run-time.

For 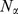 uncorrelated samples with coordinates 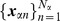 obtained from the umbrella system 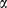 (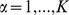), the MBAR equation defines an estimator of the free energy 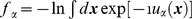 of the umbrella system 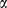 up to an additive constant [11]:
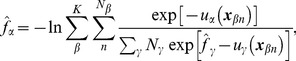
(3)where 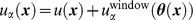 (in units of 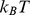) with *u* being the potential function of the system. This coupled nonlinear equation was solved by a Newton-Raphson solver [11]. The samples were subsampled prior to calculation, based on the autocorrelation function of potential energy [59]. Calculations were performed in MATLAB (The MathWorks, Inc.), confirming that the calculation was compatible with the Python implementation of the MBAR method (https://simtk.org/home/pymbar) [11].

Having obtained the free energy estimations 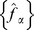, the equilibrium expectation of a mechanical observable 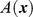 under the unrestrained ensemble can be computed as a ratio of partition functions, 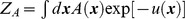 and 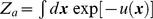:
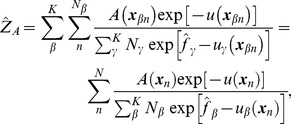
(4)

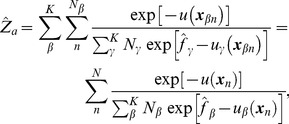
(5)where the double index of the coordinates, 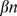, is aggregated into a single index *n* (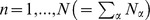), since the explicit notation of the umbrella windows (from which samples were taken) is not necessary in the remaining calculations. Equations 4 and 5 yield an estimator of the expectation 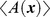:
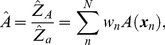
(6)where 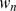 is the weight of the sample 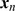 for the unrestrained equilibrium.

The probability is now assigned to an arbitrary region, designated cell *B*, by
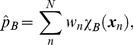
(7)where 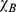 is the indicator function which takes the value 1 if the system is in the cell *B* and 0 otherwise. Knowing 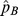, the PMF of cell *B* is given up to an additive constant in units of 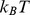:
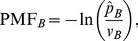
(8)where 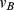 is the relative volume of cell *B*, necessary to correct for non-uniform cell size. To evaluate the PMF around the image on the string in the 20-dimensional principal component space (Figures 2A and 2B), the cells 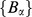 were assigned to each image 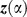 of the string using the Voronoi tessellation [9], [60], [61]:
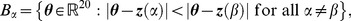
(9)where 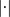 is the Euclidean distance in the 20-dimensional space. It is noted that metric tensor is not required in this definition since our collective variables are defined with linear coordinates [9], [60], [61]). During the calculation, since the explicit determination of the cell boundaries in 20-dimensional space is not feasible, the samples 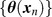 were assigned by finding their nearest images. Samples located far from any image were excluded from the calculation as outliers by imposing a cutoff condition on Equation 9, i.e., samples for which 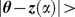
6 Å for all 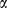 were eliminated. Increasing the cutoff distance to 7 Å did not affect the results. The relative volume 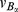 in Equation 8 was approximated by the variance of the samples in cell 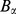, or the Voronoi cell with the cutoff condition was approximated by an ellipsoid. It was confirmed that the volume correction didn't give qualitative difference in the PMF due to the cutoff condition (see Figure S10). If the cells are spherical, a 10% error in estimating the length of the axis for each dimension roughly leads to a 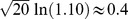 (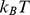) error, under the assumption that the errors are statistically independent of each other. As another way to estimate the PMF in a high-dimensional space without the Voronoi tessellation, one could perform the kernel density estimations using analytically tractable kernel functions, e.g., hyper-spheres (cutoff conditions), or Gaussian kernels. Since the volume of each kernel can be analytically calculated, this approach is free from the problem of the estimation errors in the volumes.

In the strings before attaining the MFEP (*t* = 0–10 ns), the distribution formed by the umbrella sampling tended to be biased to the minima of free energy surface, or to the MFEP. For the purpose of calculating accurate PMF for the non-converged strings (Figures 2 and S1), it was necessary that the Voronoi cells were defined by using all images generated from all strings. On the other hand, the average quantities along MFEP (Figures 3–6 and S2) were able to be correctly evaluated by using only the samples generated from the MFEP.

The RMSF values along the MFEP (Figures 3C and 3D) were evaluated with the average structure 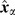 in cell 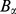 calculated by
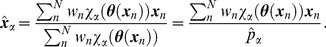
(10)The average coordinates calculated by Equation 10 were also utilized in the various investigations (Figures 5B, 6, S3, and S4). The RMSF value in cell 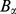 and atom *i*, 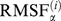, (Figures 3C, and 3D) was then evaluated by
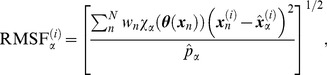
(11)where 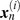 are the coordinates of atom *i* and sample *n*.

The distribution function of oxygens and hydrogens of water molecules (Figures 6A, 6B, and 6C) was evaluated as follows: First, the instantaneous densities of water atoms at grid *l* in cell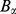, 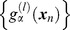, were calculated in 3D grids (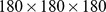) with grid size about 0.5 Å. The averaged density, 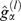, was then calculated by
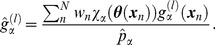
(12)


### Committor Test

In principle, the committor test launches unbiased MD simulations from structures chosen randomly from the barrier region and examines whether the resultant trajectories terminate in either the reactant or the product state with equal probability [7], [9], [33]. Here, since such a full committor test is not feasible due to the large system size, 40 unbiased MD simulations of 10 ns were started from 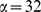, 33 or 34, that is, 120 simulations or 1.2 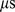 in total, and the distributions of the final structures after 10 ns were monitored [9]. Ten initial coordinates were taken randomly from the snapshots of the umbrella sampling belonging to each of Voronoi cells of 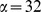, 33, or 34. Forty unrestrained MD simulations were launched from each Voronoi cell using these ten coordinates, each assigned four sets of momenta generated from the Maxwell-Boltzmann distribution. The Voronoi cell index was assigned to the coordinates after 10 ns. A histogram of the indices is plotted in Figure 5A.

## Supporting Information

Figure S1 **Statistical uncertainties of the PMFs along the strings.** Statistical uncertainties of the PMF estimations along the snapshots of the string are plotted for (A) apo and (B) holo-AK at *t* = 0 (initial path), 2, 4, 6, 8, 10, 12 ns (MFEP). Error bars indicate the statistical uncertainties relative to the PMF minimum. The uncertainties were estimated by the MBAR method. The uncertainty due to the errors in the estimate of cell volume was not counted in this plot (see *Materials and Methods* for this error).
(TIF)

Figure S2
**RMSD of the string from the initial path.** The RMSD is plotted as a function of time for apo (blue line) and holo-AK (red line), respectively. The RMSD is defined by 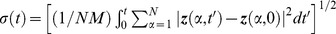, where 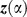 is the 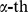 image of the string defined in the collective variable space, *M* is the number of collective variables, and *N* is the total number of images.(TIF)

Figure S3
**Rigid-body domains detected by DynDom analysis.** The averaged backbone structures along the MFEPs are shown for (A) apo and (B) holo-AK. The blue, red and yellow residues indicate the rigid-body domains detected by the DynDom program. The blue domains are fixed in space, while the red and yellow domains are moving domains which undergo rigid-body screw motions (i.e., translations and rotations). The green residues are involved in bending between the domains, and the gray residues correspond to non-rigid parts. The color of the axes of screw motion matches that of the moving domain around which the screw motion occurs. (A) In apo-AK, we performed the DynDom analysis for two sets of pairs of averaged backbone structures, 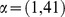, and 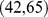. In the early stages of conformational closing (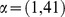), the closure of the LID domain was detected as the sole rigid-body screw motion. The closure of the AMPbd was detected at late stages, (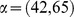). (B) In holo-AK, the analysis was performed on three sets of pairs, 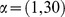, 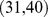, and 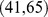. The closure of the AMPbd domain was detected in the middle stages (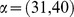), and the conformational closing is completed by the closure of the LID domain at 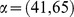. All of these results are consistent with Figs. 2Cand 2D (the projection of the MFEP onto the inter-domain distances between the LID-CORE and AMPbd-CORE domains).(TIF)

Figure S4 **Convergence of the pathway using a different initial path.** Projections of the pathways onto the space defined by the distance between the Cα mass centers of the LID-CORE and AMPbd-CORE domains for apo-AK. The black solid curve indicates the initial path (*t* = 0) different from that of the text, and the red solid curve is the path at *t* = 16.8 ns. The contour lines are same as those of Figure 2C and 2D. The initial path was created by a targeted MD simulation along the natural cubic spline that interpolates the open crystal structure, an intermediate structure, and the closed crystal structure in the 20 principal component space. The intermediate structure was created by superimposing the open and closed crystal structures. In order to create an AMPbd-first-closing initial path, we changed the weights of the superposition for each domain: LID(intermediate) = 0.7 LID(open) + 0.3 LID(closed), AMPbd(intermediate) = 0.5 AMPbd(open) + 0.5 AMPbd(closed), and CORE(intermediate) = 0.5 CORE(open) + 0.5 CORE(closed). The dashed curves represent the initial path and the MFEP of the text, respectively. The inset shows the RMSD from the MFEP of the text as a function of time.
(TIF)

Figure S5 **Comparison with a FRET experiment.** PMF as a function of the distance between the Cα atoms of Lys145 and Ile52 for apo-AK (indicated by the solid line) and holo-AK (the dashed line). These were evaluated by using the snapshots obtained from the umbrella samplings along the MFEPs.
(TIF)

Figure S6
**“Cracking” around the LID-CORE hinge and stabilization by ATP-binding.** (A) Average structure of the LID-CORE hinge for apo-AK at 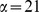. Breakage of a salt-bridge (Asp118-Lys136, represented by sticks) is shown. (B) Average structure for holo-AK at 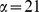. Contacts between Arg119, His134, and the ATP ribose are indicated by the dashed yellow lines.(TIF)

Figure S7 **Schematic representations of the mechanisms of the AMPbd and LID domain closures.** (A) The AMPbd domain closure matches the induced-fit mechanism; the insertion of AMP into the binding pocket first compacts the system. Additional contacts between AMP and non-hinge regions further compact the system and stabilize the compact state. (B) The LID domain closure matches the “population-shift followed by induced-fit” scenario; even in the absence of the ligand, the LID domain possesses a partially closed state which is stabilized by the “cracking” of the LID-CORE hinge (and the P-loop). The cracking of the hinge region enables rearrangement of molecular interactions for ATP-binding which induces a smooth bending of the hinge directed toward the closed conformation. As the LID closes, ATP is conveyed into the P-loop, with removal of an occluded water molecule.
(TIF)

Figure S8 **Cumulative contributions of PCs to the total variance (excluding the first).** PCA was applied to the mixed sets of two MD snapshots around the open and closed crystal structures in the absence of the ligand. The first principal mode represents the difference of the two distributions around the open and closed conformations (90% contribution). Excluding the first, the 20 PCs make 82% cumulative contribution to the total variance.
(TIF)

Figure S9 **Structures of the principal modes.** Structures of the principal modes are superimposed on the average coordinates used in the PCA. Within the 20 principal modes used in the string method, only the first six are shown. The principal modes clearly represent the collective domain motions. The first principal mode roughly corresponds to linear interpolation between the open and closed conformations.
(TIF)

Figure S10 **Effect of the volume corrections on the estimations of the PMF.** PMFs along the snapshots of the strings with the volume correction (indicated by the solid lines), and those without the volume correction (the dashed line) for (A) apo and (B) holo-AK. The line colors are same as those of Fig. 2A and 2B.
(TIF)

Video S1 **Average structures along the MFEP for apo-AK.**
(MOV)

Video S2 **Average structures along the MFEP for holo-AK.**
(MOV)
