# Supplementary figures and images for: Minimum Free Energy Path of Ligand-Induced Transition in Adenylate Kinase
Source: PLoS Comput Biol. 2012 Jun 7;8(6):e1002555. doi: 10.1371/journal.pcbi.1002555 (PMC3369945; doi:10.1371/journal.pcbi.1002555)

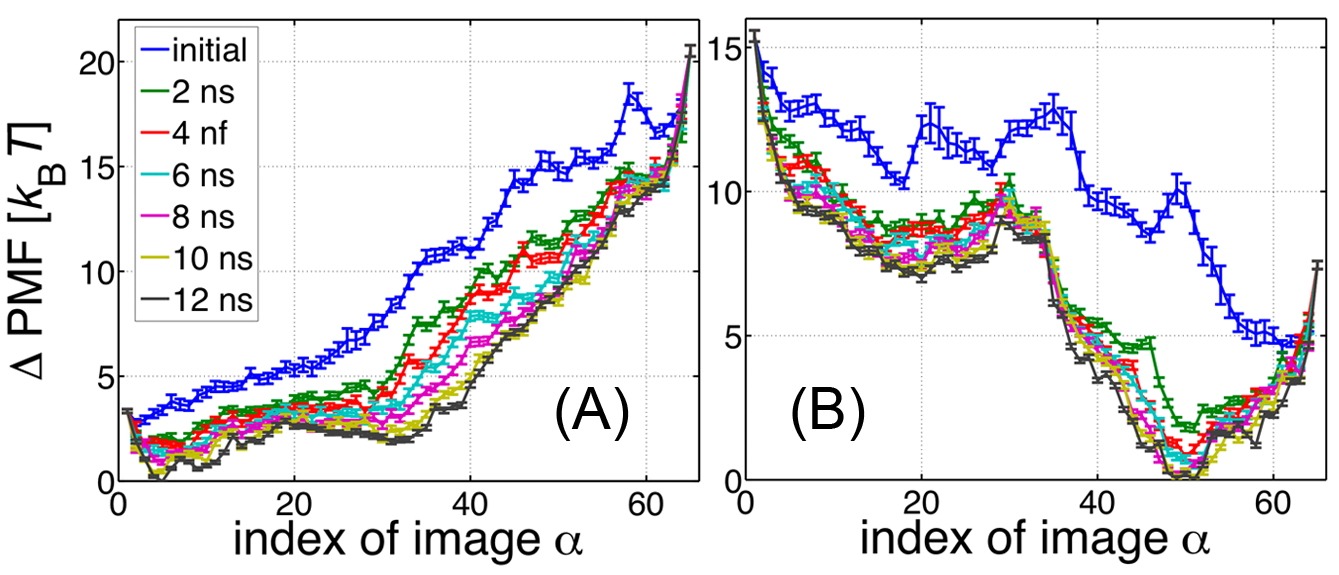

Supplement: Figure S1 — Statistical uncertainties of the PMFs along the strings. Statistical uncertainties of the PMF estimations along the snapshots of the string are plotted for (A) apo and (B) holo-AK at t = 0 (initial path), 2, 4, 6, 8, 10, 12 ns (MFEP). Error bars indicate the statistical uncertainties relative to the PMF minimum. The uncertainties were estimated by the MBAR method. The uncertainty due to the errors in the estimate of cell volume was not counted in this plot (see Materials and Methods for this error). (TIF) [file pcbi.1002555.s001.tif]

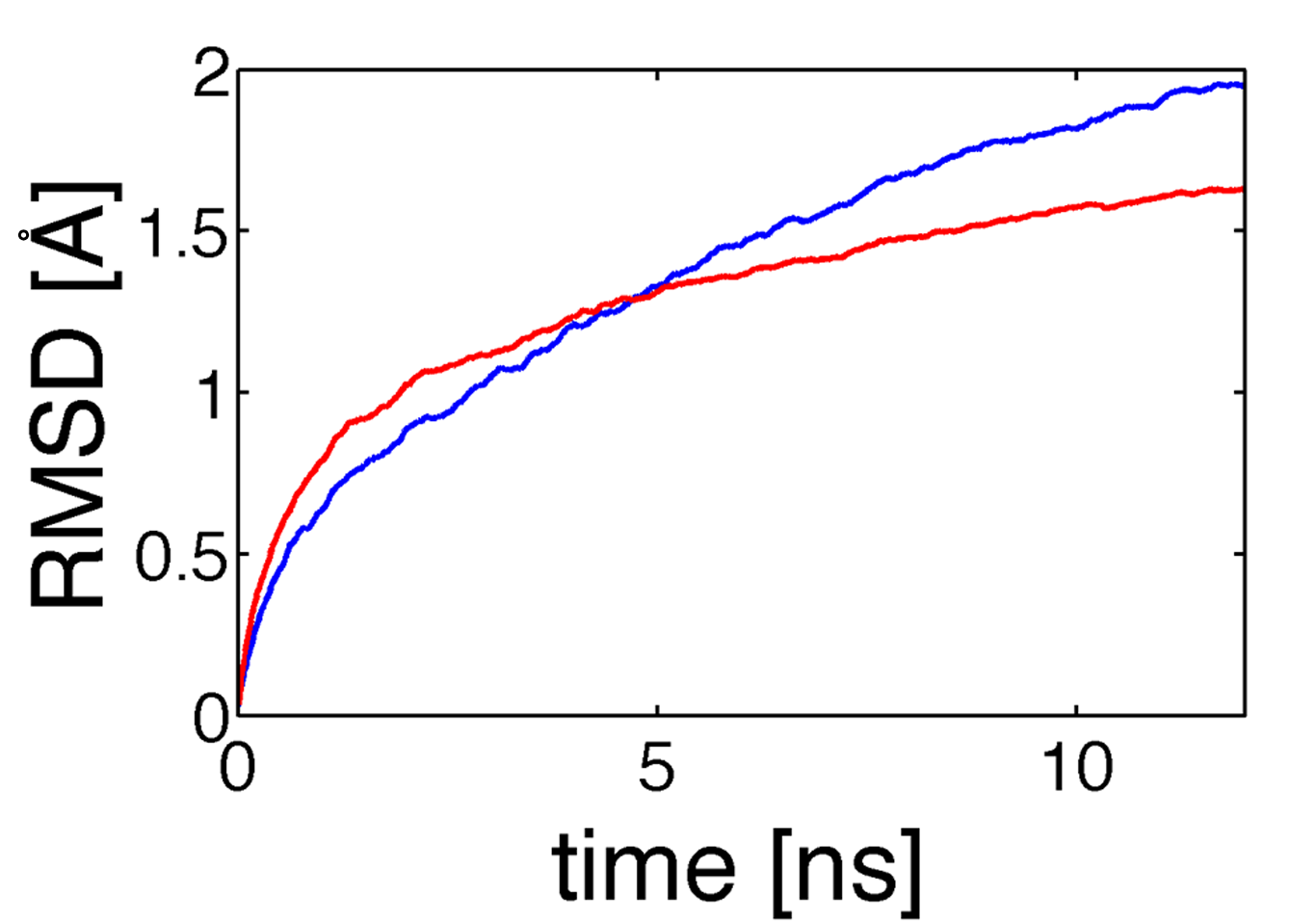

Supplement: Figure S2 — RMSD of the string from the initial path. The RMSD is plotted as a function of time for apo (blue line) and holo-AK (red line), respectively. The RMSD is defined by , where is the image of the string defined in the collective variable space, M is the number of collective variables, and N is the total number of images. (TIF) [file pcbi.1002555.s002.tif]

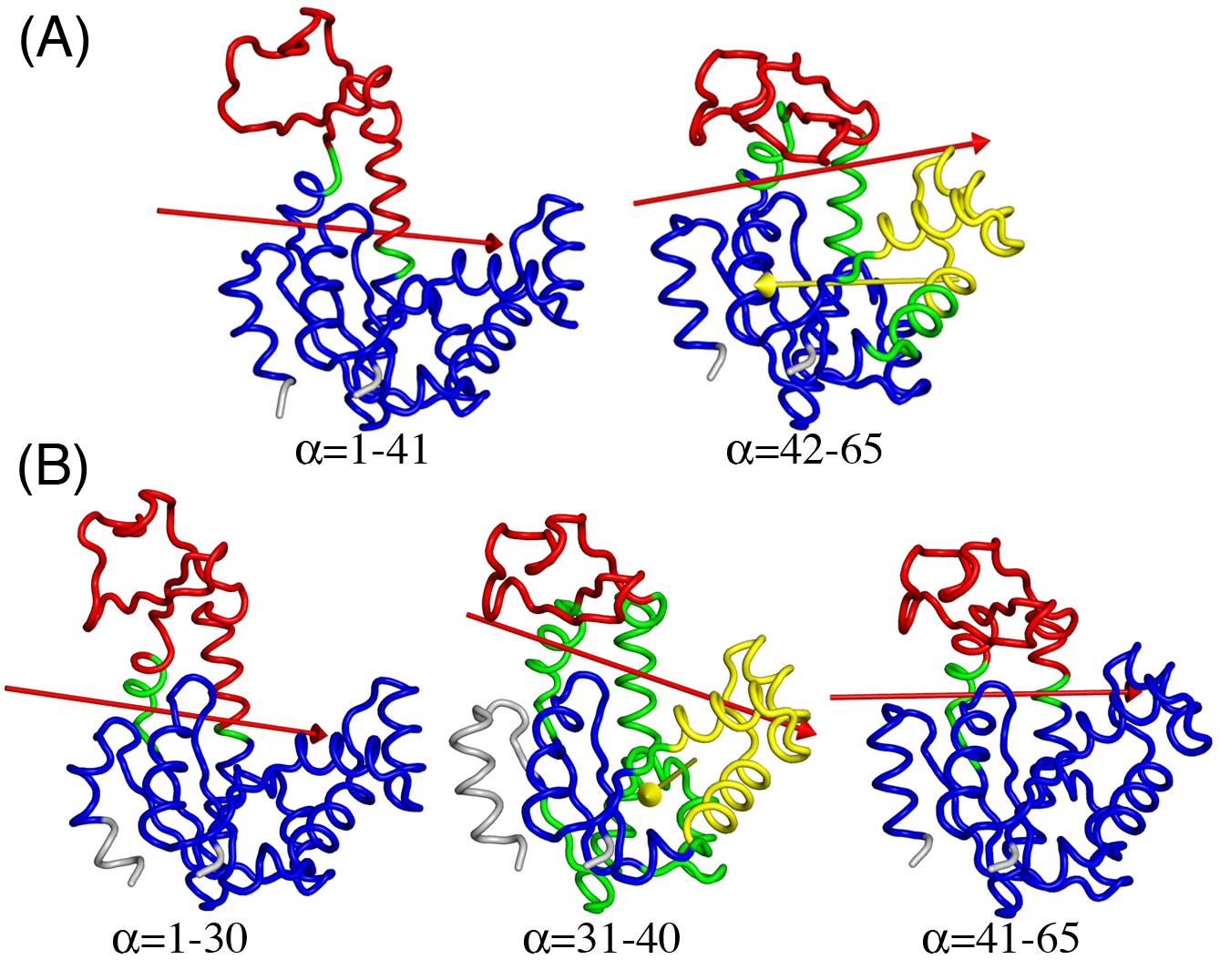

Supplement: Figure S3 — Rigid-body domains detected by DynDom analysis. The averaged backbone structures along the MFEPs are shown for (A) apo and (B) holo-AK. The blue, red and yellow residues indicate the rigid-body domains detected by the DynDom program. The blue domains are fixed in space, while the red and yellow domains are moving domains which undergo rigid-body screw motions (i.e., translations and rotations). The green residues are involved in bending between the domains, and the gray residues correspond to non-rigid parts. The color of the axes of screw motion matches that of the moving domain around which the screw motion occurs. (A) In apo-AK, we performed the DynDom analysis for two sets of pairs of averaged backbone structures, , and . In the early stages of conformational closing (), the closure of the LID domain was detected as the sole rigid-body screw motion. The closure of the AMPbd was detected at late stages, (). (B) In holo-AK, the analysis was performed on three sets of pairs, , , and . The closure of the AMPbd domain was detected in the middle stages (), and the conformational closing is completed by the closure of the LID domain at . All of these results are consistent with Figs. 2Cand 2D (the projection of the MFEP onto the inter-domain distances between the LID-CORE and AMPbd-CORE domains). (TIF) [file pcbi.1002555.s003.tif]

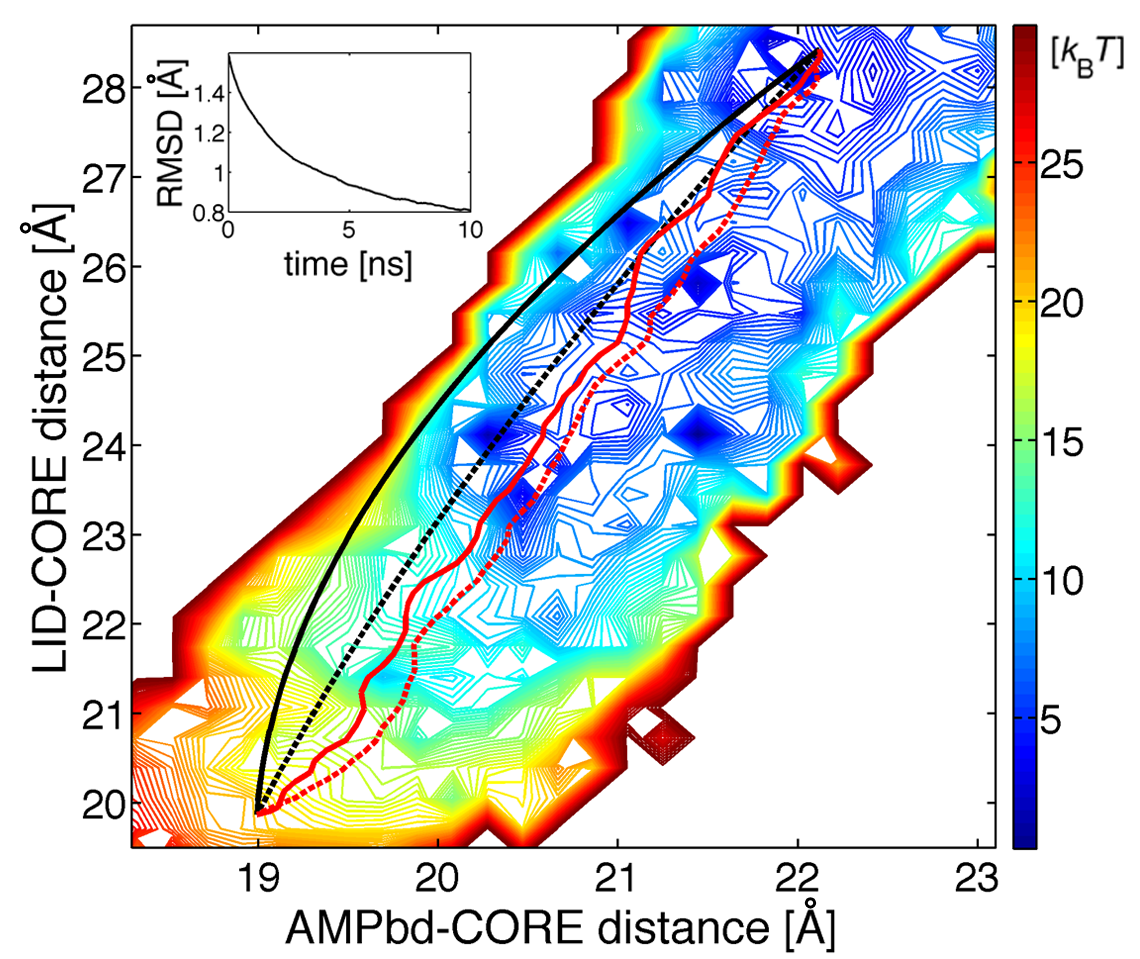

Supplement: Figure S4 — Convergence of the pathway using a different initial path. Projections of the pathways onto the space defined by the distance between the Cα mass centers of the LID-CORE and AMPbd-CORE domains for apo-AK. The black solid curve indicates the initial path (t = 0) different from that of the text, and the red solid curve is the path at t = 16.8 ns. The contour lines are same as those of Figure 2C and 2D. The initial path was created by a targeted MD simulation along the natural cubic spline that interpolates the open crystal structure, an intermediate structure, and the closed crystal structure in the 20 principal component space. The intermediate structure was created by superimposing the open and closed crystal structures. In order to create an AMPbd-first-closing initial path, we changed the weights of the superposition for each domain: LID(intermediate) = 0.7 LID(open) + 0.3 LID(closed), AMPbd(intermediate) = 0.5 AMPbd(open) + 0.5 AMPbd(closed), and CORE(intermediate) = 0.5 CORE(open) + 0.5 CORE(closed). The dashed curves represent the initial path and the MFEP of the text, respectively. The inset shows the RMSD from the MFEP of the text as a function of time. (TIF) [file pcbi.1002555.s004.tif]

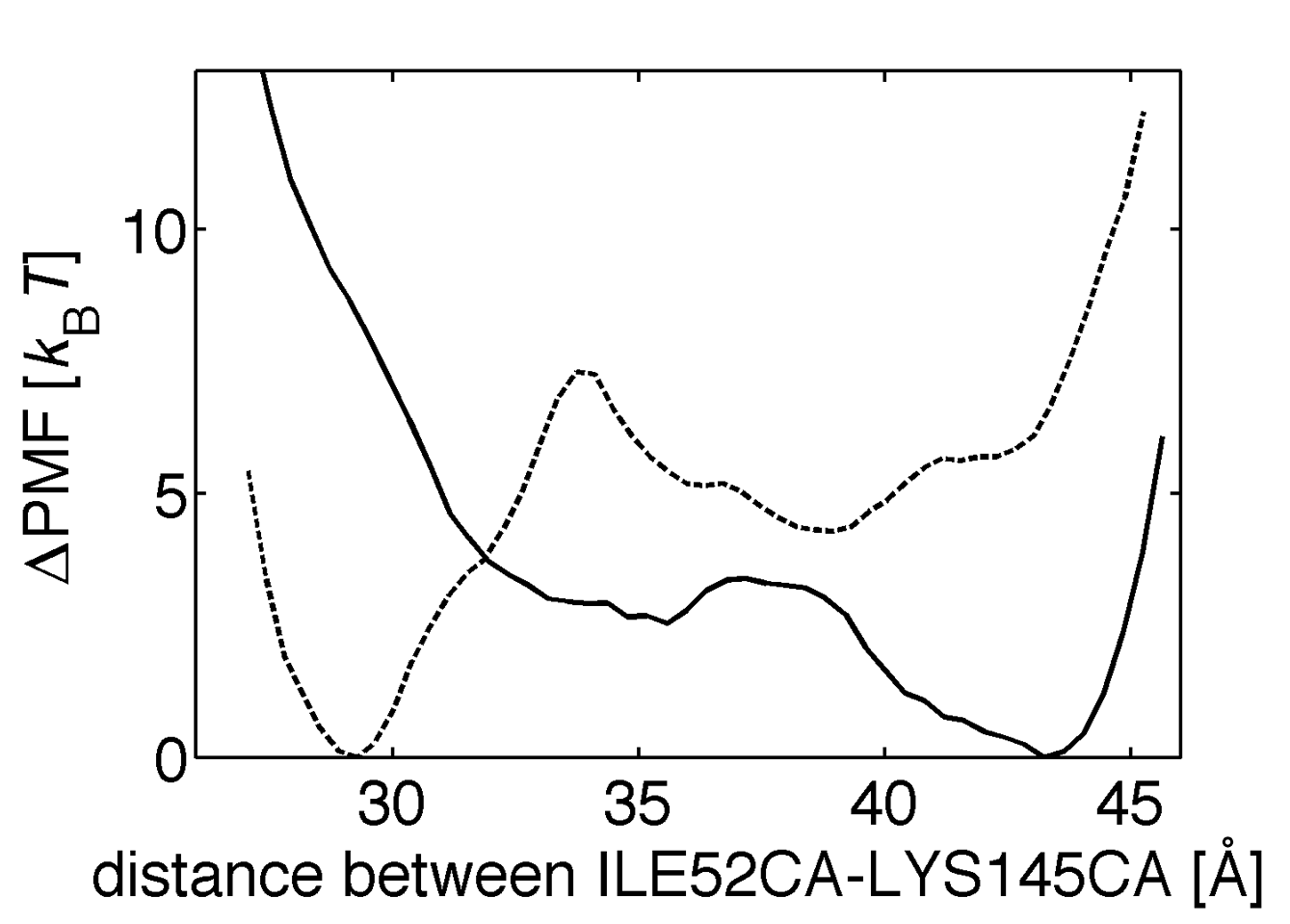

Supplement: Figure S5 — Comparison with a FRET experiment. PMF as a function of the distance between the Cα atoms of Lys145 and Ile52 for apo-AK (indicated by the solid line) and holo-AK (the dashed line). These were evaluated by using the snapshots obtained from the umbrella samplings along the MFEPs. (TIF) [file pcbi.1002555.s005.tif]

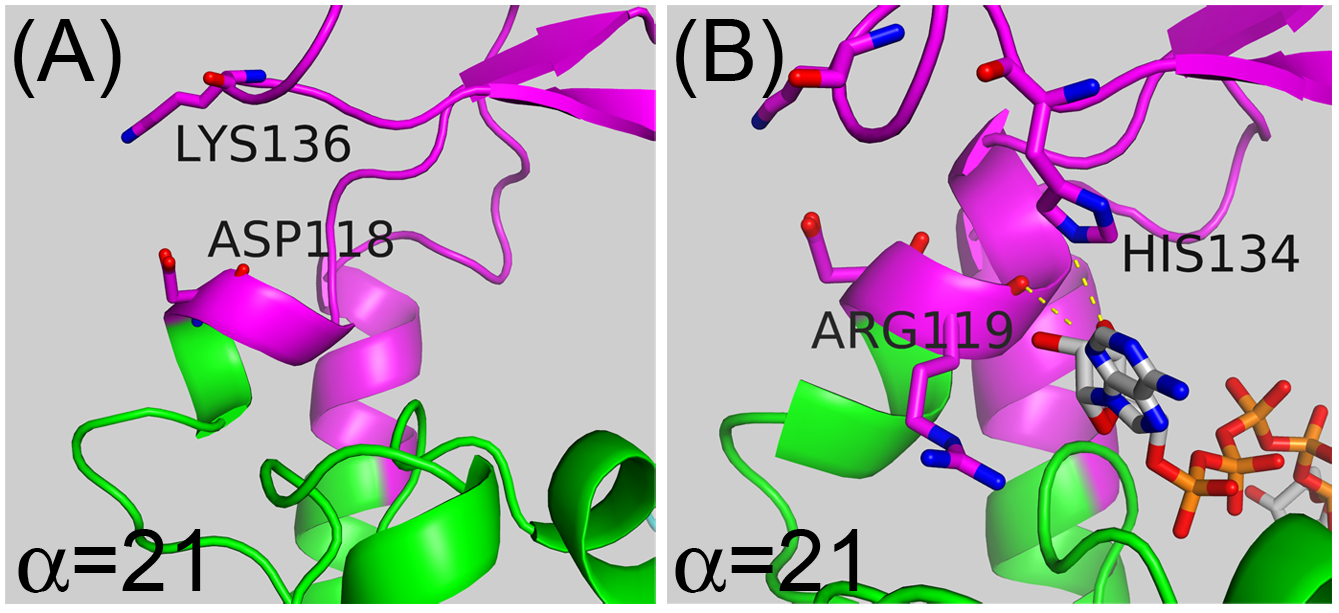

Supplement: Figure S6 — “Cracking” around the LID-CORE hinge and stabilization by ATP-binding. (A) Average structure of the LID-CORE hinge for apo-AK at . Breakage of a salt-bridge (Asp118-Lys136, represented by sticks) is shown. (B) Average structure for holo-AK at . Contacts between Arg119, His134, and the ATP ribose are indicated by the dashed yellow lines. (TIF) [file pcbi.1002555.s006.tif]

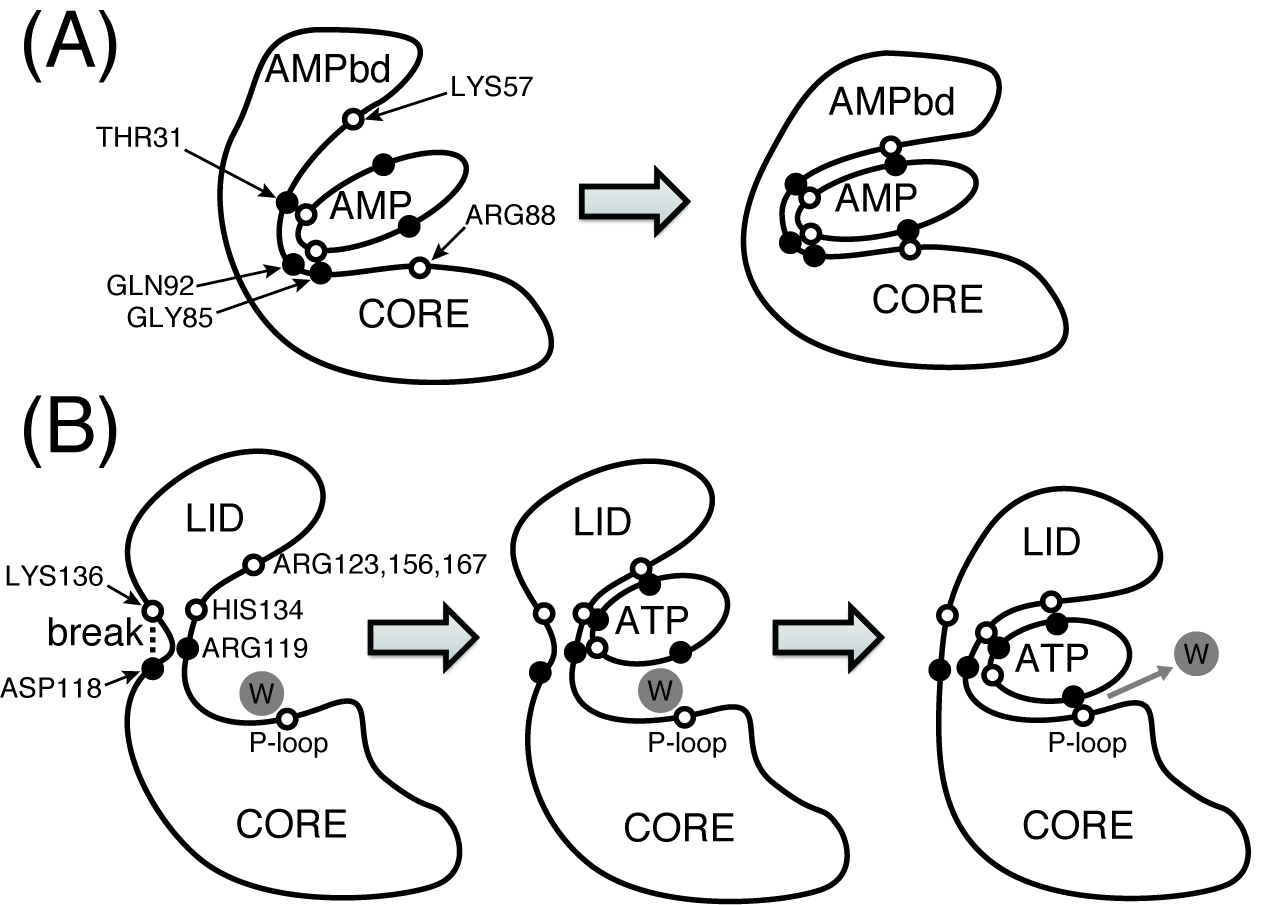

Supplement: Figure S7 — Schematic representations of the mechanisms of the AMPbd and LID domain closures. (A) The AMPbd domain closure matches the induced-fit mechanism; the insertion of AMP into the binding pocket first compacts the system. Additional contacts between AMP and non-hinge regions further compact the system and stabilize the compact state. (B) The LID domain closure matches the “population-shift followed by induced-fit” scenario; even in the absence of the ligand, the LID domain possesses a partially closed state which is stabilized by the “cracking” of the LID-CORE hinge (and the P-loop). The cracking of the hinge region enables rearrangement of molecular interactions for ATP-binding which induces a smooth bending of the hinge directed toward the closed conformation. As the LID closes, ATP is conveyed into the P-loop, with removal of an occluded water molecule. (TIF) [file pcbi.1002555.s007.tif]

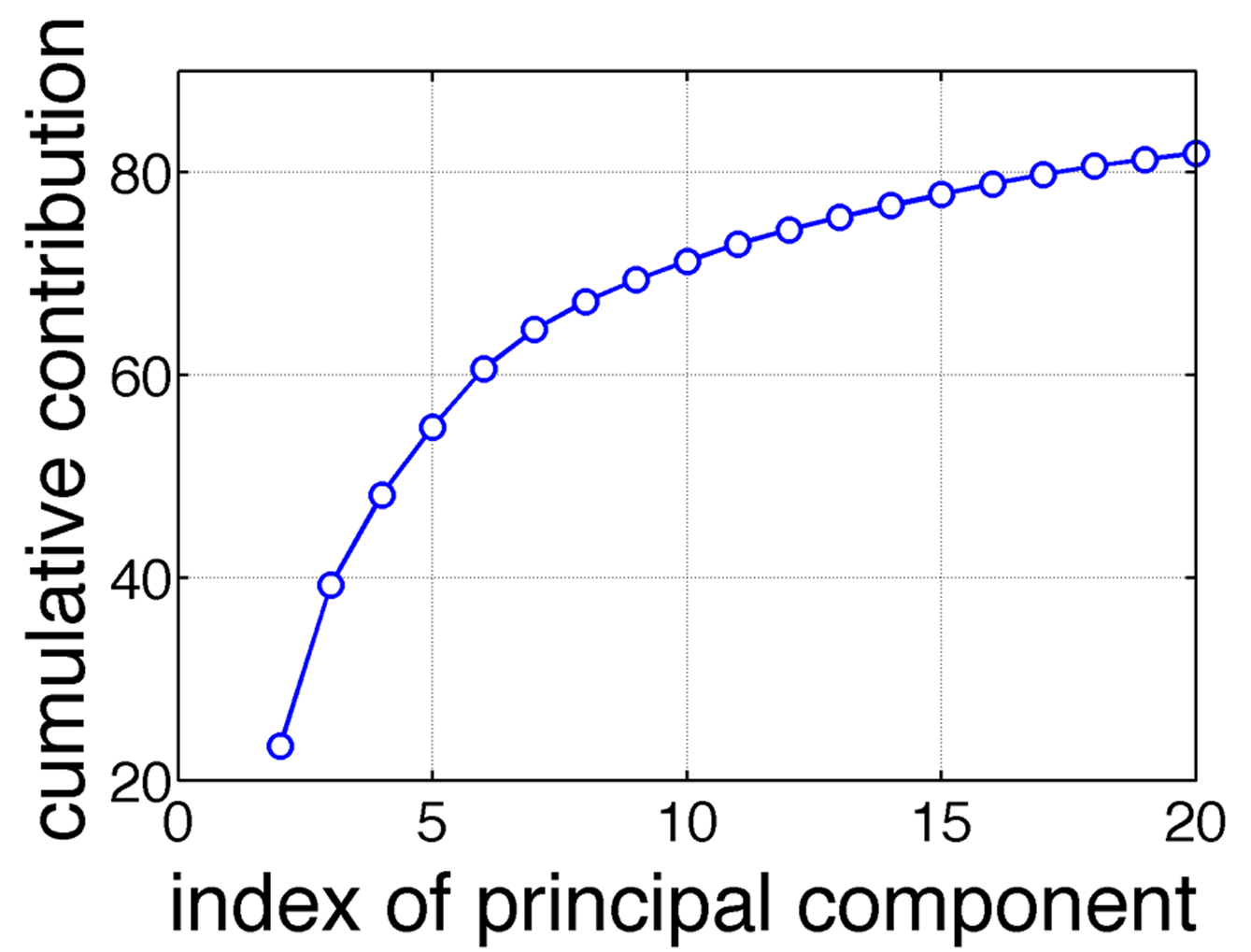

Supplement: Figure S8 — Cumulative contributions of PCs to the total variance (excluding the first). PCA was applied to the mixed sets of two MD snapshots around the open and closed crystal structures in the absence of the ligand. The first principal mode represents the difference of the two distributions around the open and closed conformations (90% contribution). Excluding the first, the 20 PCs make 82% cumulative contribution to the total variance. (TIF) [file pcbi.1002555.s008.tif]

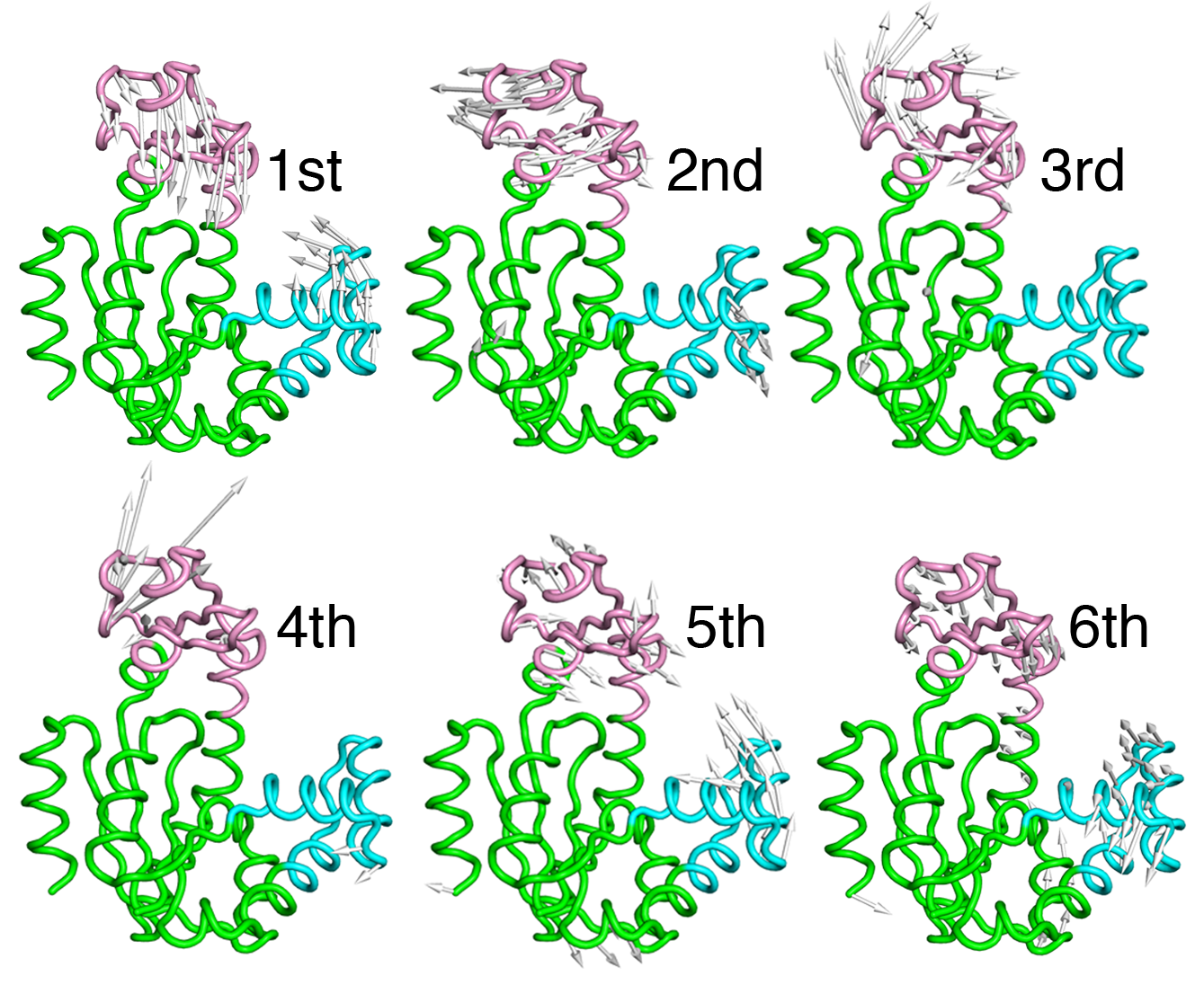

Supplement: Figure S9 — Structures of the principal modes. Structures of the principal modes are superimposed on the average coordinates used in the PCA. Within the 20 principal modes used in the string method, only the first six are shown. The principal modes clearly represent the collective domain motions. The first principal mode roughly corresponds to linear interpolation between the open and closed conformations. (TIF) [file pcbi.1002555.s009.tif]

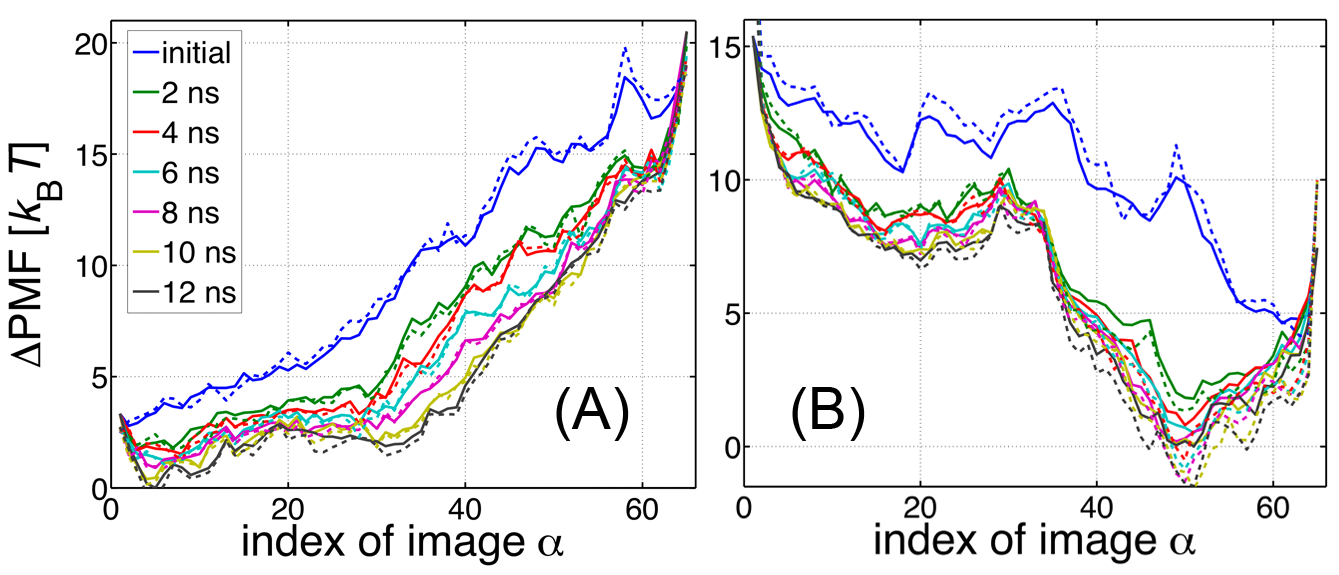

Supplement: Figure S10 — Effect of the volume corrections on the estimations of the PMF. PMFs along the snapshots of the strings with the volume correction (indicated by the solid lines), and those without the volume correction (the dashed line) for (A) apo and (B) holo-AK. The line colors are same as those of Fig. 2A and 2B. (TIF) [file pcbi.1002555.s010.tif]
